# Secular trends in the prevalence of abdominal obesity among Chinese adults with normal weight, 1993–2015

**DOI:** 10.1038/s41598-021-95777-y

**Published:** 2021-08-12

**Authors:** Xingxing Sun, Zhelong Liu, Tingting Du

**Affiliations:** 1 Department of Anesthesiology, Tongji Hospital, Tongji Medical College, Huazhong University of Science and Technology, Wuhan, Hubei 430030 People’s Republic of China; 2 Department of Endocrinology, Tongji Hospital, Tongji Medical College, Huazhong University of Science and Technology, Wuhan, Hubei 430030 People’s Republic of China; 3 Branch of National Clinical Research Center for Metabolic Diseases, Wuhan, Hubei People’s Republic of China

**Keywords:** Epidemiology, Obesity

## Abstract

A considerable chronic disease burden existed in people with normal body mass index (BMI), it is imperative to study the prevailing trends in abdominal obesity among Chinese people with normal BMI. Hence, we aimed to analyze updated prevalence data on abdominal obesity trends among Chinese adults with a normal BMI. We used data from the China Health and Nutrition Survey (CHNS) conducted between 1993 and 2015. Abdominal obesity is defined as waist circumference (WC) ≥ 90 cm for men and ≥ 80 cm for women following the International Diabetes Federation recommendations for Asians. Over the 23-year period, the age-standardized mean WC values showed a significant increasing trend among Chinese adults with BMI < 25 kg/m^2^, with the mean value increased from 74.0 cm to 78.5 cm (P for trend < 0.0001). During the period of 1993–2015, the age-standardized prevalence of abdominal obesity increased from 12.1 to 26.0% (P for trend < 0.0001). Significant increases were observed in both sexes, all age groups, rural and urban residents, and all educational attainment groups (all P for trends < 0.0001), with a greater relative increase noted among men, younger participants, and rural residents. Similar significant trends were noted when a more stringent BMI < 23 kg/m^2^ cut point (Asian cut point) was applied. A low magnitude of overlap existed between abdominal obesity and general obesity, irrespective of the criteria used. The mean WC and the prevalence of abdominal obesity among Chinese adults with normal BMI increased continuously from 1993 to 2015. The upward trends were noted in both sexes, all age groups, rural and urban regions, and all educational attainment groups. Our estimates emphasize the importance of adding WC in addition to BMI as measures to monitor obesity prevalence.

## Introduction

With the dramatic shift in diet from traditional to western dietary patterns and a steep decline in physical activity levels that have followed the rapid urbanization and industrialization, increasing rates of obesity in China are especially alarming^1,2^. China has moved from 60th place for men and 41st place for women in 1975 to second for both men and women in 2014 in the worldwide ranking of the number of severely obese individuals^3^. Approximately one in five obesity individuals worldwide are Chinese^2^. The fast increase in the prevalence of obesity in China has been accompanied by marked increases in an expanding set of chronic diseases, including diabetes mellitus, cardiovascular disease, chronic kidney disease, many cancers, and an array of cognitive and musculoskeletal disorders^4^.

Normally, body mass index (BMI) has been used as a proxy for obesity in the population. However, abdominal obesity assessed by waist circumference (WC) is more pathogenic and thus is more closely associated with type 2 diabetes, cardiovascular disease, and cancer mortality than general obesity^5–8^. Further, BMI is a poor indicator of body fat distribution, as evidenced by the occurrence of the variation in the burden of diabetes mellitus, and cardiovascular disease among individuals with similar BMI^9–11^. In addition, type 2 diabetes, and cardiovascular disease are not rare diseases in persons with normal BMI^12^. For example, 7.6% of Chinese individuals with normal BMI suffered from diabetes^9,12^, more than 20% of the normal weight population encountered with metabolic disorders^9^, and globally, 39% of deaths and 37% of disability-adjusted life-years occurred among nonobese persons^4^. Taken together, it is important to know the trends in abdominal obesity. Although trends in abdominal obesity in China have been evaluated in several studies^1,13^, very few studies describe the trends in abdominal obesity among individuals with normal BMI^14^. Given that Chinese population, who, despite being generally less obese, are more prone to visceral fat accumulation compared with western populations^15^, the increase in WC was more pronounced than the increase in BMI at given periods in China^13^, and that a considerable disease burden existed in people with normal BMI, it is imperative to study the prevailing trends in abdominal obesity among Chinese people with normal BMI. Our previous study reported an upward trend from 1993 to 2009 in the prevalence of abdominal obesity among Chinese people with normal BMI irrespective of sex, age, rural/urban settings, and education levels^14^. It is hypothesized that analyses of data from recent years will show further increases. To get a more comprehensive understanding of the trends in abdominal obesity among Chinese people with normal BMI over the years from 2009 to 2015, this study presents new data for recent trends in abdominal obesity from 2009 to 2015.

## Methods

### Study design

We used data from the China Health and Nutrition Survey (CHNS) for our analysis. The CHNS is a series of cross-sectional household-based surveys conducted by the Carolina Population Center at the University of North Carolina at Chapel Hill and the National Institute of Nutrition and Food Safety at the Chinese Center for Disease Control and Prevention. Full details of the study have been described elsewhere^1,14,16^. Briefly, the CHNS rounds were conducted in 1989, 1991, 1993, 1997, 2000, 2004, 2006, 2009, 2011, 2015, and 2019. A stratified multistage, random cluster sampling was employed to draw study sample from 12 provinces (Liaoning, Heilongjiang, Shandong, Shaanxi, Henan, Hubei, Hunan, Guangxi, Guizhou, Jiangsu, Yunnan and, Zhejiang), that vary significantly in terms of geography, economic development, and health status. The survey procedures were reviewed and approved by the institutional review committees of the University of North Carolina at Chapel Hill, and the National Institute of Nutrition and Food Safety, Chinese Center for Disease Control and Prevention in accordance with the ethical standards laid down in the 1964 Declaration of Helsinki and its later amendments. Informed consent was obtained from all participants and/or their legal guardians.

### Study population

All participants were asked to complete a structured questionnaire which provided information on age, sex, place of residence, educational attainment, and medical history. Participants were included in the present analysis if they were 18 years or older. Exclusion criteria included pregnancy, and with missing information on age, WC, BMI, or blood pressure (BP), and with extreme or implausible WC (51 cm or 190 cm), BMI, or BP values. The remaining analytic sample sizes for the current study were 7745 in 1993, 8351 in 1997, 9331 in 2000, 8993 in 2004, 8856 in 2006, 9338 in 2009, 12,539 in 2011, and 11,259 in 2015.

### Measurements

Weight, height, WC and BP were measured following standardized protocols from the World Health Organization (WHO)^17,18^. WC was initially collected in 1993. Weight was measured with the participants wearing light clothing on a calibrated beam scale and height was measured without shoes using a portable stadiometer. BMI was calculated as weight in kilograms divided by height in meters squared. WC was measured with an inelastic tape to the nearest 0.1 cm at a midpoint between the bottom of the rib cage and the top of the iliac crest at the end of exhalation. BP was measured by trained technicians in triplicate after a 10-min rest, using mercury manometers. The three readings were averaged as the BP values in our data analysis. All physical examinations were performed at the same location and followed the same protocol at each study visit.

### Definitions

According to WHO suggestions^18^, normal weight is defined as BMI < 25 kg/m^2^, general obesity is defined as BMI ≥ 30 kg/m^2^. According to WHO expert consultation for Asians^19^, normal weight is defined as BMI < 23 kg/m^2^. According to the criteria recommended by Working Group on Obesity in China^20^, general obesity is defined as BMI ≥ 28 kg/m^2^. In the present study, we referred normal weight defined by BMI < 25 kg/m^2^ and obesity defined by BMI ≥ 30 kg/m^2^ as the broad criteria, and normal weight defined by BMI < 23 kg/m^2^ and obesity defined by BMI ≥ 28 kg/m^2^ as the stringent criteria. According to the International Diabetes Federation recommendations for Asians^21^, abdominal obesity is defined as WC ≥ 90 cm for men and ≥ 80 cm for women.

### Statistical analysis

All statistical analyses were performed with SAS version 9.2 (SAS Institute Inc., Cary, North Carolina). Continuous variables were presented as means and standard deviations (SD). Categorical variables were expressed as numbers or percentages. One-way ANOVA was applied to compare differences in means across groups. A Chi-square test was performed to assess differences of proportions across groups. Bonferroni correction was applied to adjust P-values for multiple comparisons. To take into account the changes in population age structure during the survey period, the estimated mean WC and prevalence of abdominal obesity were age-standardized to the 2010 census of the Chinese adult population by the direct method. Time trends for the mean WC across the surveys were assessed using linear regression models, with the year of the survey entered as a continuous variable. Trends in the prevalence of central obesity among participants with BMI < 25 kg/m^2^ from 1993 to 2015 were assessed by logistic regression models. In the trend analysis, age, sex, region, and educational attainment were adjusted in the linear or logistic regression models except when used as a stratified variable. A sensitivity analysis was performed using a more stringent BMI < 23 kg/m^2^ cut point (Asian cut point). To assess whether changes throughout the 23-year period differed by sex, logistic regression analysis was utilized to examine potential interaction effects between cohort and sex. Similar processes were repeated separately for age groups, rural/urban regions, and educational attainment groups. Venn diagram was constructed as a visual display of how abdominal obesity defined by WC and general obesity defined by BMI clustered together. A two-tailed *P* value of < 0.05 was considered to be statistically significant.

## Results

The mean age of the study population increased progressively from 1993 to 2015 (P < 0.0001) (Supplementary Table 1). The percentage of women increased progressively from 1997 to 2011, and then has a slight decrease in 2015. Over time, the proportion of older adults increased and the proportion of younger adults decreased; The proportion of respondents from urban declined; The educational attainment increased notably; Characteristics of the sample with normal BMI from each survey showed similar patterns (Supplementary Table 2).

Trends in the age-standardized mean WC from 1993 to 2015 among Chinese adults with BMI < 25 kg/m^2^ were shown for overall and by sex, age, region categories, and educational attainment in Table 1. Overall, age-standardized mean WC increased by 4.5 cm, (P for trend < 0.0001). The increase in the age-standardized mean WC in men was greater than that in women (P < 0.0001 for interaction terms survey × sex), with the age-standardized mean WC in men increased from 75.1 cm in 1993 to 81.1 cm in 2015, and in women from 73.1 cm in 1993 to 76.6 cm in 2015. For each survey, the age-specific mean WC increased with age (all P for trend < 0.0001). Further, for each age group, the age-standardized mean WC increased linearly from 1993 to 2015 (P < 0.0001). The age-standardized mean WC increased over time in both rural and urban regions and in all educational attainment categories (all P for trends < 0.0001). Notably, mean WC increased more in rural versus urban regions (P < 0.0001 for interaction terms survey × rural/urban regions), and more in less than high school education group (P = 0.0015 for interaction terms survey × educational attainment groups).

**Table 1:** Trends in age-standardized mean waist circumference among Chinese adults with body mass index < 25 kg/m^2^, 1993–2015.

	1993	1997	2000	2004	2006	2009	2011	2015	*P for trend	^⁑^P for trend	P for interaction	^†^P (1993 vs 2015)	^‡^P (1993 vs 2015)
**Total**	74.0 ± 7.5	74.9 ± 7.5	76.1 ± 8.0	76.9 ± 8.1	77.0 ± 8.0	77.7 ± 8.3	78.3 ± 9.1	78.5 ± 11.1	< 0.0001	< 0.0001		< 0.0001	< 0.0001
Men	75.1 ± 7.2	76.4 ± 7.3	77.7 ± 7.8	78.9 ± 8.0	79.0 ± 7.8	79.6 ± 8.1	80.7 ± 9.0	81.1 ± 10.4	< 0.0001	< 0.0001	< 0.0001	< 0.0001	< 0.0001
Women	73.1 ± 7.6	73.5 ± 7.4	74.6 ± 7.9	74.9 ± 7.9	75.2 ± 7.9	76.0 ± 8.3	76.4 ± 8.8	76.6 ± 11.3	< 0.0001	< 0.0001		< 0.0001	< 0.0001
**Age (years)**													
18–44	72.7 ± 6.9	74.0 ± 7.0	74.8 ± 7.5	75.9 ± 7.8	76.0 ± 7.8	76.4 ± 8.1	77.2 ± 8.9	77.3 ± 10.9	< 0.0001	< 0.0001	0.7613	< 0.0001	< 0.0001
45–64	75.7 ± 7.6	76.2 ± 7.5	78.0 ± 7.7	78.8 ± 7.7	79.0 ± 7.5	80.1 ± 7.9	80.5 ± 9.0	80.6 ± 10.5	< 0.0001	< 0.0001		< 0.0001	< 0.0001
65–118	76.1 ± 9.0	76.5 ± 8.9	78.6 ± 9.3	78.5 ± 9.1	79.3 ± 8.8	80.4 ± 8.7	80.7 ± 9.2	80.7 ± 11.7	< 0.0001	< 0.0001		< 0.0001	< 0.0001
**Region**													
Urban	75.0 ± 8.4	75.1 ± 7.9	76.2 ± 8.6	77.0 ± 8.5	77.3 ± 8.3	77.5 ± 8.4	78.2 ± 9.2	78.5 ± 12.1	< 0.0001	< 0.0001	< 0.0001	< 0.0001	< 0.0001
Rural	73.6 ± 7.0	74.8 ± 7.3	76.1 ± 7.7	76.7 ± 7.9	76.8 ± 7.9	77.7 ± 8.3	78.3 ± 9.1	78.4 ± 10.4	< 0.0001	< 0.0001		< 0.0001	< 0.0001
**Education**													
Less than high school	74.8 ± 7.5	75.4 ± 7.6	77.0 ± 7.9	77.4 ± 8.0	77.6 ± 8.0	78.8 ± 8.4	79.1 ± 9.3	79.2 ± 10.8	< 0.0001	< 0.0001	0.0015	< 0.0001	< 0.0001
High school	74.2 ± 7.1	75.1 ± 7.2	76.1 ± 7.6	76.8 ± 8.0	76.9 ± 7.7	77.4 ± 8.1	77.8 ± 9.0	77.9 ± 10.7	< 0.0001	< 0.0001		< 0.0001	< 0.0001
University	74.3 ± 8.0	75.1 ± 7.8	75.9 ± 8.6	76.5 ± 8.4	76.5 ± 8.4	76.9 ± 8.5	77.6 ± 9.0	77.7 ± 11.7	< 0.0001	< 0.0001		< 0.0001	< 0.0001

Mean WC were age-standardized to the age distribution of the China population in 2010.

*P value for trend analysis. Linear regression model was used without any adjustment in the model.

^⁑^P value for trend analysis in which age, sex, region, and educational attainment were adjusted in the linear models except when used as a stratified variable.

^†^P value for comparison of 1993 and 2015. One-way ANOVA was used to compare differences. Applying the Bonferroni method for adjusting for multiple comparisons. There were 28 implied comparisons and an α = 0.0018 (α = 0.05/28) was used.

^‡^P value for comparison of 1993 and 2015 after adjusting for age, sex, region, and educational attainment except when used as a stratified variable.

Although with relatively lower WC values (Table 2), the trends in WC from 1993 to 2015 were similar among Chinese adults with BMI < 23 kg/m^2^ (Asian cut point for normal weight).

**Table 2:** Trends in age-standardized mean waist circumference among Chinese adults with body mass index < 23 kg/m^2^, 1993–2015.

	1993	1997	2000	2004	2006	2009	2011	2015	*P for trend	^⁑^P for trend	P for interaction	^†^P (1993 vs 2015)	^‡^P (1993 vs 2015)
**Total**	72.6 ± 6.8	73.5 ± 6.9	74.4 ± 7.2	75.0 ± 7.6	75.0 ± 7.4	75.5 ± 7.7	75.9 ± 8.5	76.2 ± 10.7	< 0.0001	< 0.0001		< 0.0001	< 0.0001
Men	73.8 ± 6.5	75.0 ± 6.6	75.9 ± 7.0	76.7 ± 7.4	77.0 ± 7.2	77.3 ± 7.5	78.2 ± 8.6	78.2 ± 10.1	< 0.0001	< 0.0001	< 0.0001	< 0.0001	< 0.0001
Women	71.5 ± 6.8	72.0 ± 6.8	72.9 ± 7.2	73.3 ± 7.4	73.3 ± 7.2	74.1 ± 7.7	74.4 ± 8.1	74.5 ± 10.8	< 0.0001	< 0.0001		< 0.0001	< 0.0001
**Age (years)**													
18–44	71.5 ± 6.3	72.8 ± 6.5	73.3 ± 6.9	74.0 ± 7.3	74.2 ± 7.3	74.3 ± 7.3	74.9 ± 8.2	74.9 ± 10.4	< 0.0001	< 0.0001	0.0163	< 0.0001	< 0.0001
45–64	74.1 ± 6.9	74.3 ± 6.8	75.7 ± 7.0	76.5 ± 7.3	76.5 ± 6.8	77.5 ± 7.4	77.9 ± 8.4	77.8 ± 10.3	< 0.0001	< 0.0001		< 0.0001	< 0.0001
65–118	74.3 ± 8.1	74.7 ± 8.1	76.2 ± 8.4	76.2 ± 8.4	76.9 ± 8.1	78.1 ± 8.1	78.1 ± 8.8	78.1 ± 11.1	< 0.0001	< 0.0001		< 0.0001	< 0.0001
**Region**													
Urban	73.3 ± 7.8	73.5 ± 7.3	74.5 ± 7.9	75.0 ± 8.0	75.2 ± 7.7	75.3 ± 7.8	76.0 ± 8.9	76.1 ± 11.1	< 0.0001	< 0.0001	0.2655	< 0.0001	< 0.0001
Rural	72.3 ± 6.3	73.5 ± 6.6	74.3 ± 6.9	74.9 ± 7.4	74.9 ± 7.2	75.6 ± 7.6	75.9 ± 8.3	75.9 ± 10.4	< 0.0001	< 0.0001		< 0.0001	< 0.0001
**Education**													
Less than high school	73.2 ± 6.7	73.9 ± 6.9	75.0 ± 7.2	75.3 ± 7.4	75.4 ± 7.3	76.5 ± 7.7	76.8 ± 8.7	76.9 ± 10.6	< 0.0001	< 0.0001	0.0006	< 0.0001	< 0.0001
High school	73.0 ± 6.6	73.8 ± 6.7	74.3 ± 6.8	75.0 ± 7.6	75.1 ± 7.0	75.2 ± 7.5	75.5 ± 8.5	75.7 ± 10.5	< 0.0001	< 0.0001		< 0.0001	< 0.0001
University	72.9 ± 7.3	73.2 ± 6.9	74.3 ± 8.1	74.7 ± 8.0	74.5 ± 7.8	74.9 ± 7.8	75.4 ± 8.4	75.2 ± 10.9	< 0.0001	< 0.0001		< 0.0001	< 0.0001

Mean WC were age-standardized to the age distribution of the China population in 2010.

*P value for trend analysis. Linear regression model was used without any adjustment in the model.

^⁑^P value for trend analysis in which age, sex, region, and educational attainment were adjusted in the linear models except when used as a stratified variable.

^†^P value for comparison of 1993 and 2015. One-way ANOVA was used to compare differences. Applying the Bonferroni method for adjusting for multiple comparisons. There were 28 implied comparisons and an α = 0.0018 (α = 0.05/28) was used.

^‡^P value for comparison of 1993 and 2015 after adjusting for age, sex, region, and educational attainment except when used as a stratified variable.

**Figure 1:**
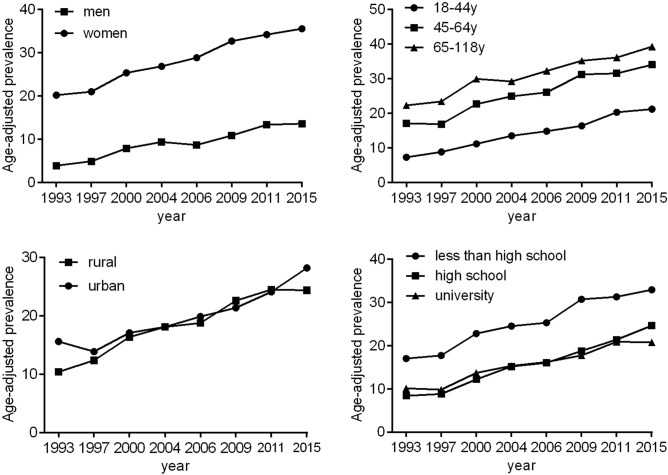
Trends in the age-adjusted prevalence of abdominal obesity among Chinese adults with body mass index < 25 kg/m^2^ by sex, age, rural/urban setting, and educational attainment: the CHNS 1993–2015. The estimated prevalence of abdominal obesity was age-standardized to the age distribution of the China population in 2010 by the direct method. The corresponding statistics and P values were showed in the Supplementary Table 3.

In 1993, the age-standardized prevalence of abdominal obesity among Chinese adults with BMI < 25 kg/m^2^ was 12.1%. By 2015, the prevalence was more than doubled. Prevalence of abdominal obesity increased faster in men, from 3.9% in 1993 to 13.6% in 2015 in men, and from 20.2% in 1993 to 35.6% in 2015 in women (Fig. 1 and Supplementary Table S3). The prevalence of abdominal obesity increased significantly between 1993 and 2015 in all studied subgroups (all P for trends < 0.0001) (Fig. 1 and Supplementary Table 3). Of note, trends in the prevalence of abdominal obesity from 1993 to 2015 increased more in younger participants than that in those aged ≥ 65 years (P < 0.0001 for interaction terms survey × age), and in rural than that in urban regions (P = 0.0001 for interaction terms survey × rural/urban regions).

**Figure 2:**
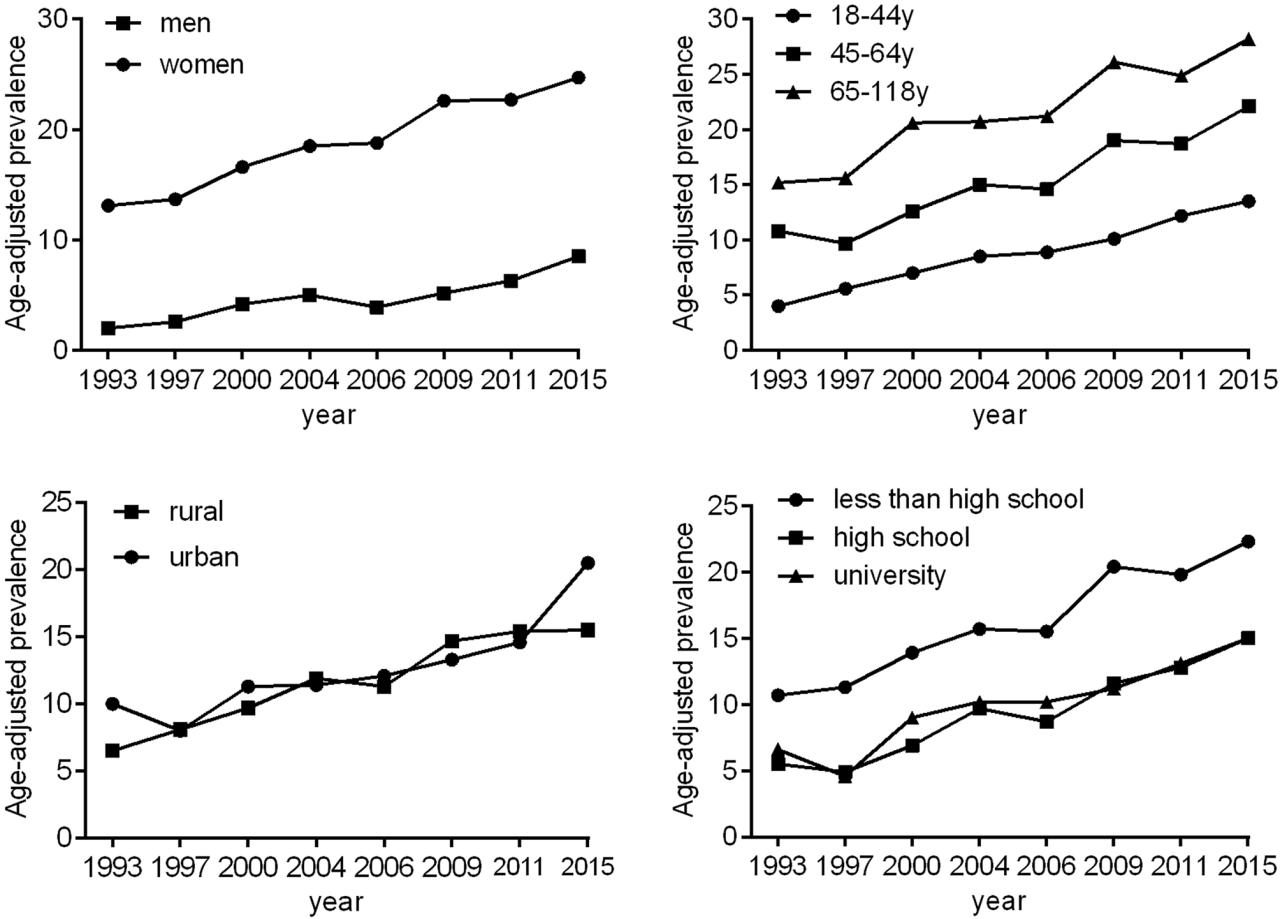
Trends in the age-adjusted prevalence of abdominal obesity among Chinese adults with body mass index < 23 kg/m^2^ by sex, age, rural/urban setting, and educational attainment: the CHNS 1993–2015. The estimated prevalence of abdominal obesity was age-standardized to the age distribution of the China population in 2010. The corresponding statistics and P values were showed in the Supplementary Table 4.

The prevalence of abdominal obesity also worsened overtime when a more stringent BMI < 23 kg/m^2^ cut point was applied to define normal weight (Fig. 2 and Supplementary Table 4).

Trend analysis showed similar patterns when age, sex, region, and education attainment were adjusted in the linear or logistic regression models (Tables 1, 2, Supplementary Tables 3, 4).

How abdominal obesity defined by WC ≥ 90 cm in men and 80 cm in women and general obesity defined by BMI ≥ 30 kg/m^2^ clustered together for each survey was showed in Table 3. For each survey, among those subjects with either abdominal obesity or general obesity, considerable participants (more than 86.4%) had exclusive abdominal obesity compared with very rare (less than 1.5%) with exclusive general obesity. A low magnitude of overlap (less than 13%) existed between abdominal obesity and general obesity. Exclusive general obesity was even less frequent in women than in men.

**Table 3:** Overlap between body mass index (≥ 30 kg/m^2^)- and waist circumference-based obesity among Chinese adults.

	1993		1997		2000		2004		2006		2009		2011		2015	
	n	%	n	%	n	%	n	%	n	%	n	%	n	%	n	%
**Total**																
BMI ≥ 30 kg/m^2^ or WC ≥ 90/80 cm	1495	–	2014	–	2918	–	3210	–	3359	–	4007	–	5988	–	5971	–
Exclusive BMI ≥ 30 kg/m^2^	4	0.3	2	0.1	8	0.3	6	0.2	11	0.3	9	0.2	56	0.9	26	0.4
Exclusive WC ≥ 90/80 cm	1421	95.1	1864	92.6	2684	92.0	2914	90.8	3053	90.9	3627	90.5	5305	88.6	5264	88.2
Both BMI ≥ 30 kg/m^2^and WC ≥ 90/80 cm	70	4.7	148	7.3	226	7.7	290	9.0	295	8.8	371	9.3	627	10.5	681	11.4
**Men**																
BMI ≥ 30 kg/m^2^ or WC ≥ 90/80 cm	332	–	596	–	924	–	1021	–	1072	–	1350	–	2171	–	2121	–
Exclusive BMI ≥ 30 kg/m^2^	2	0.6	1	0.2	4	0.4	2	0.2	9	0.8	6	0.4	32	1.5	13	0.6
Exclusive WC ≥ 90/80 cm	308	92.8	544	91.3	832	90.0	919	90.0	947	88.3	1185	87.8	1876	86.4	1833	86.4
Both BMI ≥ 30 kg/m^2^and WC ≥ 90/80 cm	22	6.6	51	8.6	88	9.5	100	9.8	116	10.8	159	11.8	263	12.1	275	13.0
**Women**																
BMI ≥ 30 kg/m^2^ or WC ≥ 90/80 cm	1163	–	1418	–	1994	–	2189	–	2287	–	2657	–	3817	–	3850	–
Exclusive BMI ≥ 30 kg/m^2^	2	0.2	1	0.1	4	0.2	4	0.2	2	0.1	3	0.1	24	0.6	13	0.3
Exclusive WC ≥ 90/80 cm	1113	95.7	1320	93.1	1852	92.9	1995	91.1	2106	92.1	2442	91.9	3429	89.8	3431	89.1
Both BMI ≥ 30 kg/m^2^ and WC ≥ 90/80 cm	48	4.1	97	6.8	138	6.9	190	8.7	179	7.8	212	8.0	364	9.5	406	10.5

Proportions of obesity based on both WC ≥ 90/80 cm and BMI ≥ 30 kg/m^2^ (overlap between BMI- and WC-based obesity) is calculated as the number of obesity based on both WC ≥ 90/80 cm and BMI ≥ 30 kg/m^2^ divided by the number of obesity based on either WC ≥ 90/80 cm or BMI ≥ 30 kg/m^2^.

Likewise, proportions of exclusive general obesity and exclusive central obesity are calculated by the similar procedure.

Overlap results were shown in Table 4 when using a more stringent BMI ≥ 28 kg/m^2^ cut point was applied to define general obesity. For each survey, among those subjects with either abdominal obesity or general obesity, considerable participants (more than 66%) had exclusive abdominal obesity compared with very rare (less than 3.8%) with exclusive general obesity. A low magnitude of overlap (less than 31%) existed between abdominal obesity and general obesity. Exclusive general obesity was even less frequent in women than in men.

**Table 4:** Overlap between body mass index (≥ 28 kg/m^2^)- and waist circumference-based obesity among Chinese adults.

	1993		1997		2000		2004		2006		2009		2011		2015	
	n	%	n	%	n	%	n	%	n	%	n	%	n	%	n	%
**Total**																
BMI ≥ 28 kg/m^2^ or WC ≥ 90/80 cm	1513	–	2035	–	2949	–	3241	–	3399	–	4033	–	6034	–	6023	–
Exclusive BMI ≥ 28 kg/m^2^	22	1.5	23	1.1	39	1.3	37	1.1	51	1.5	35	0.9	102	1.7	78	1.3
Exclusive WC ≥ 90/80 cm	1272	84.1	1610	79.1	2319	78.6	2527	78.0	2665	78.4	3145	78.0	4527	75.0	4392	72.9
Both BMI ≥ 28 kg/m^2^and WC ≥ 90/80 cm	219	14.5	402	19.8	591	20.0	677	20.9	683	20.1	853	21.2	1405	23.3	1553	25.8
**Men**																
BMI ≥ 28 kg/m^2^ or WC ≥ 90/80 cm	343	–	612	–	948	–	1046	–	1102	–	1372	–	2207	–	2170	–
Exclusive BMI ≥ 28 kg/m^2^	13	3.8	17	2.8	28	3.0	27	2.6	39	3.5	28	2.0	68	3.1	62	2.9
Exclusive WC ≥ 90/80 cm	264	77.0	428	69.9	679	71.6	755	72.2	800	72.6	990	72.2	1536	69.6	1435	66.1
Both BMI ≥ 28 kg/m^2^and WC ≥ 90/80 cm	66	19.2	167	27.3	241	25.4	264	25.2	263	23.9	354	25.8	603	27.3	673	31.0
**Women**																
BMI ≥ 28 kg/m^2^ or WC ≥ 90/80 cm	1170	–	1423	–	2001	–	2195	–	2297	–	2661	–	3827	–	3853	–
Exclusive BMI ≥ 28 kg/m^2^	9	0.8	6	0.4	11	0.5	10	0.5	12	0.5	7	0.3	34	0.9	16	0.4
Exclusive WC ≥ 90/80 cm	1008	86.2	1182	83.1	1640	82.0	1772	80.7	1865	81.2	2155	81.0	2991	78.2	2957	76.7
Both BMI ≥ 28 kg/m^2^ and WC ≥ 90/80 cm	153	13.1	235	16.5	350	17.5	413	18.8	420	18.3	499	18.8	802	21.0	880	22.8

Proportions of obesity based on both WC ≥ 90/80 cm and BMI ≥ 28 kg/m^2^ (overlap between BMI- and WC-based obesity) is calculated as the number of obesity based on both WC ≥ 90/80 cm and BMI ≥ 28 kg/m^2^ divided by the number of obesity based on either WC ≥ 90/80 cm or BMI ≥ 28 kg/m^2^. Likewise, proportions of exclusive general obesity and exclusive central obesity are calculated by the similar procedure.

## Discussion

The present study, in which the most recent data from CHNS, including the 2015 survey, were analyzed, showed that mean WC and the prevalence of abdominal obesity have increased dramatically among Chinese adults with normal weight, irrespective of which criterion was used, since 1993. The increases occurred in both genders, all age groups, rural and urban residents, and all educational attainment groups. Moreover, men, younger participants, and rural residents showed relatively faster increases. Our results are of particular concern as abdominal obesity, which reasonably represents visceral adiposity, is closely associated with obesity-related conditions and mortality in those with a normal BMI^8^.

Although few studies have analyzed trends in mean WC and prevalence of abdominal obesity among people with acceptable BMI, our results are generally in line with those of previous studies showing that a considerable proportion of individuals suffered from abdominal obesity among people with normal BMI^22^. Further, emerging evidence showed that WC increased much faster than BMI during the same periods, and hence the relative increase in abdominal obesity at the same periods is much larger than that of general obesity^23,24^. Our previous study showed a dramatic upward trend in the prevalence of abdominal obesity among people with normal BMI from 1993 to 2009^14^. The present study showed that the increasing trend from 2009 to 2015 appeared to continue rather than slow or level off. Our results together with previous reports suggest that body composition has changed over time. Evidence showed that approximately 20% higher risk of mortality occurred in individuals with normal BMI who were abdominally obese compared with their counterparts with normal BMI who were not abdominally obese^25^ and that the association between WC and mortality was strongest in those with a normal BMI^8^. Thus, depicting the changing trend of abdominal obesity among people with acceptable BMI may provide additional information for more accurately assessing the prevalence of obesity-related disorders. Considering the more deleterious effect of visceral fat on metabolic disorders than subcutaneous fat^26–29^, the increase in WC is likely to be due to a relatively greater increase in visceral adipose tissue than that of subcutaneous fat. Therefore, it is urgent to take interventional strategies to reverse abdominal obesity trends and reduce the likely medical costs of the increase in abdominal obesity in normal-weight people.

The continuing rapid increase in the prevalence of abdominal obesity among individuals with normal BMI in China from 1993 to 2015 is attributed to several factors. Unhealthy lifestyles and behavioral changes are probably major drivers. Increased availability, accessibility, and affordability of energy-dense foods and a more sedentary lifestyle that have followed urbanization and increasingly mechanized transportation and labor are responsible for excess energy intake and reduced energy expenditure, respectively, and thus induce fat accumulation in the body. For example, China has been experiencing westernization of their diet. As a result, the consumption of plant-based foods such as cereals and starchy roots has dramatically declined; in contrast, the intake of foods rich in sugar, fat, and refined carbohydrates as well as animal-based food such as red meat, and processed meat has dramatically increased^30^. Active transportation, such as walking or cycling, which was associated with a decrease in obesity and weight gain, covered up to 80% of daily travel in China until the 1990s, but this situation declined dramatically thereafter^31,32^. TV ownership increased dramatically during the recent two decades, with 38 sets per 1,000 persons in 1985 and 112 to 135 sets per 100 households in 2011^33^. Future unfavorable trends in dietary pattern and physical activity level will exacerbate the increase in the prevalence of abdominal obesity.

Our findings that the rates of such an increase in abdominal obesity prevalence varied by sex, age, and rural/urban regions are also a characteristic noted in other studies^1,23,34^. Although the root causes that induce the difference is not clear, disparities between subgroups in genetic, sociocultural, socioeconomic, and behavioral factors, such as disparities in calorie intake, knowledge and means to adopt healthy lifestyles as well as weight management programs, and mechanized transport and work, have been considered as potential drivers^30^. Despite the inequalities in the abdominal obesity prevalence by sex, age, and rural/urban regions, our study showed that the abdominal obesity prevalence increased over time in all subgroups, indicating a leading role of the obesogenic environment in China in the recent two decades. Further, our finding that the continuous increase in the prevalence of abdominal obesity was much faster in younger people is concerning because it predicts the prevalence of abdominal obesity prevalence among normal-weight people should keep increasing in the next few years.

In the present study, a significant dissociation between general obesity defined by BMI and abdominal obesity defined by WC was noted. When using the stringent criterion to define general obesity, the magnitude of overlap between general obesity and abdominal obesity is 31%. Further, approximately two-thirds of individuals with obesity would be missed if WC is not taken into account for the identification of obesity. When using the broad criterion to define general obesity, a significant dissociation between general obesity (defined by the) and abdominal obesity defined by WC was noted, with only 13% with the presence of both general obesity and abdominal obesity occurring together. Further, more than 80% of individuals with obesity would be missed if WC is not taken into account for the identification of obesity. Hence, the absence of a WC measurement might result in substantial misclassification of individuals who are actually in a risk category based on WC as being in a low-risk category based on an acceptable range of BMI. Indeed, our previous study together with other reports evidenced that more than 20% of the normal weight population encountered an increased WC and a cluster of cardiovascular risk factors, including insulin resistance, atherogenic lipid profiles, hypertension, and non-acholic fatty liver disease^9^. Since increased WC and the mentioned cardiovascular risk factors exhibited increased incidences of diabetes, cardiovascular diseases, and all-cause mortality^35–38^, the stringent criteria should be used in combination with WC in the future in China to assess whether an individual was actually in a risk state and thus could provide an opportunity for proper intervention.

Our study has several strengths. First, it maintains a large sample size and includes individuals from diverse and representative regions in China, which allows for exploring the prevalence of obesity over a range of demographic groups. Second, all study measurements are made by trained staff following a standard protocol. A vigorous quality assurance program and the same sampling and strict methodology are used to ensure the quality of the data collection over the entire study period, allowing direct comparisons of results over time. Third, we assessed the secular trends in abdominal obesity in people with normal BMI, using different cut-off points for BMI and identified consistent associations. However, the limitations of the present study require careful consideration. Firstly, the sample is partial nationally representative, therefore the generalizability of the results to regions not studied may be limited. Secondly, other social and environmental variables such as dietary habits, sleep duration, and physical activity, which would have an impact on obesity, were not considered. Third, measurements of body fatness were not available.

## Conclusions

Mean WC and the prevalence of abdominal obesity increased significantly among Chinese adults with normal BMI from 1993 to 2015. The increasing trends were observed in both genders, all age groups, both rural and urban regions, and all educational attainment groups. Men, younger participants, and rural residents experienced greater increases. Our findings underline the necessary to investigate and monitor the trends in the prevalence of abdominal obesity among normal-weight individuals to improve awareness, alert health care professionals, and provide guidance for future disease prevention and health promotion.

## Supplementary Information

Supplementary Information.

## Abbreviations

BMI: Body mass index
WC: Waist circumference
CHNS: China Health and Nutrition Survey
BP: Blood pressure
WHO: World Health Organization
SD: Standard deviation
