# Supplementary material for: Secular trends in the prevalence of abdominal obesity among Chinese adults with normal weight, 1993–2015
Source: Sci Rep. 2021 Aug 12;11:16404. doi: 10.1038/s41598-021-95777-y (PMC8360975; doi:10.1038/s41598-021-95777-y)
Supplement: Supplementary file 1 — Supplementary Information. [file 41598_2021_95777_MOESM1_ESM.docx]

Secular trends in the prevalence of abdominal obesity among Chinese adults with normal weight, 1993-2015

Xingxing Sun^1^, Zhelong liu^2,3,†^ Tingting Du^2,3,†^

^1^ Department of Anesthesiology, Tongji Hospital, Tongji Medical College, Huazhong University of Science and Technology, Wuhan 430030, PR China.

^2^ Department of Endocrinology, Tongji Hospital, Tongji Medical College, Huazhong University of Science and Technology, Wuhan 430030, PR China.

^3^ Branch of national clinical research center for metabolic diseases, Hubei.

^†^Corresponding author: Tingting Du, MD, PhD, Department of Endocrinology, Tongji Hospital, Tongji Medical College, Huazhong University of Science and Technology, Wuhan, Hubei 430030, China. Branch of national clinical research center for metabolic diseases, Hubei. Tel and Fax: 86-27-83661425 E-mail: [aduttsxx@163.com](mailto:aduttsxx@163.com);

Zhelong liu, Department of Endocrinology, Tongji Hospital, Tongji Medical College, Huazhong University of Science and Technology, Wuhan, Hubei 430030, China. Branch of national clinical research center for metabolic diseases, Hubei. Tel and Fax: 86-27-83661425 E-mail: liuzhelong@163.com

**Supplementary Table 1 Unadjusted demographic characteristics of Chinese adults: the CHNS 1993–2015**

|  | 1993 | 1997 | 2000 | 2004 | 2006 | 2009 | 2011 | 2015 | P for trend |
| --- | --- | --- | --- | --- | --- | --- | --- | --- | --- |
| **N** | 7745 | 8351 | 9331 | 8993 | 8856 | 9338 | 12539 | 11259 |  |
| Women (%) | 52.3 | 51.3 | 52.1 | 52.2 | 52.9 | 52.4 | 53.0 | 51.3 | <0.0001 |
| Age (y, Mean ± SD) | 42.8 ± 15.7 | 43.4 ± 15.8 | 44.9 ± 15.5 | 47.9 ± 15.3 | 49.2 ± 15.2 | 50.3 ± 15.3 | 51.1±15.2 | 51.5±14.8 | <0.0001 |
| Age distribution (%) |  |  |  |  |  |  |  |  |  |
| 18-44 | 61.0 | 56.0 | 51.1 | 42.9 | 41.0 | 36.4 | 33.8 | 27.3 | <0.0001 |
| 45-64 | 29.1 | 32.2 | 36.1 | 41.1 | 41.7 | 45.1 | 47.1 | 48.7 | <0.0001 |
| ≥65 | 9.9 | 11.8 | 12.8 | 16.0 | 17.3 | 18.6 | 19.1 | 24.0 | <0.0001 |
| Region (%) |  |  |  |  |  |  |  |  |  |
| Rural | 31.3 | 35.8 | 34.0 | 35.3 | 34.3 | 33.4 | 41.0 | 39.2 | <0.0001 |
| Urban | 68.7 | 64.2 | 66.0 | 64.7 | 65.7 | 66.6 | 59.0 | 60.8 | <0.0001 |
| Education (%) |  |  |  |  |  |  |  |  |  |
| Less than high school | 55.2 | 56.5 | 55.5 | 42.8 | 41.7 | 40.7 | 34.1 | 31.7 | <0.0001 |
| High school | 28.8 | 26.7 | 26.6 | 32.2 | 31.1 | 34.3 | 33.2 | 33.9 | <0.0001 |
| University | 16.0 | 16.8 | 17.9 | 25.0 | 27.2 | 25.0 | 32.7 | 34.4 | <0.0001 |

**Supplementary Table 2 Unadjusted demographic characteristics of Chinese adults with people with BMI<25 kg/m^2^: the CHNS 1993–2015**

|  | 1993 | 1997 | 2000 | 2004 | 2006 | 2009 | 2011 | 2015 | P for trend |
| --- | --- | --- | --- | --- | --- | --- | --- | --- | --- |
| N | 6680 | 6824 | 7183 | 6654 | 6449 | 6608 | 8315 | 6769 |  |
| Women (%) | 50.9 | 50.3 | 51.3 | 51.3 | 52.7 | 52.4 | 53.6 | 52.8 | <0.0001 |
| Age (y, Mean ± SD) | 41.1±15.9 | 42.6±16.1 | 44.0±15.9 | 47.1±15.8 | 48.6±15.8 | 49.7±16.0 | 50.6±16.0 | 51.8±15.5 | <0.0001 |
| Age distribution (%) |  |  |  |  |  |  |  |  |  |
| 18-44 | 63.3 | 58.7 | 53.4 | 45.7 | 43.7 | 38.9 | 36.4 | 30.2 | <0.0001 |
| 45-64 | 27.0 | 29.7 | 34.0 | 38.4 | 39.0 | 42.3 | 43.7 | 45.6 | <0.0001 |
| ≥65 | 9.7 | 11.6 | 12.6 | 15.9 | 17.3 | 18.7 | 19.9 | 24.2 | <0.0001 |
| Region (%) |  |  |  |  |  |  |  |  |  |
| Rural | 29.8 | 33.8 | 32.0 | 33.4 | 33.2 | 32.9 | 40.6 | 38.9 | <0.0001 |
| Urban | 70.2 | 66.2 | 68.0 | 66.6 | 66.9 | 67.1 | 59.4 | 61.1 | <0.0001 |
| Education (%) |  |  |  |  |  |  |  |  |  |
| Less than high school | 54.6 | 56.5 | 55.3 | 42.6 | 41.5 | 40.5 | 34.4 | 31.5 | <0.0001 |
| High school | 29.8 | 27.5 | 27.5 | 32.9 | 31.1 | 34.1 | 32.2 | 33.4 | <0.0001 |
| University | 15.6 | 16.1 | 17.2 | 24.6 | 27.4 | 25.4 | 33.4 | 35.2 | <0.0001 |

**Supplementary Table 3 Age-standardized prevalence of central obesity among Chinese adults with BMI < 25 kg/m^2^**

|  | 1993 | 1997 | 2000 | 2004 | 2006 | 2009 | 2011 | 2015 | *P for trend | ⁑P for trend | P for interaction | †P (1993 vs 2015) | ‡P (1993 vs 2015) |
| --- | --- | --- | --- | --- | --- | --- | --- | --- | --- | --- | --- | --- | --- |
|  | % (SE) | % (SE) | % (SE) | % (SE) | % (SE) | % (SE) | % (SE) | % (SE) |  |  |  |  |  |
| Total | 12.1 (0.4) | 13.0 (0.4) | 16.7 (0.5) | 18.3 (0.5) | 19.2 (0.5) | 22.2 (0.6) | 24.5 (0.6) | 26.0 (0.7) | <0.0001 | <0.0001 |  | <0.0001 | <0.0001 |
| Men | 3.9 (0.4) | 4.9 (0.4) | 7.9 (0.5) | 9.4 (0.6) | 8.7 (0.5) | 10.9 (0.6) | 13.4 (0.6) | 13.6 (0.9) | <0.0001 | <0.0001 | 0.0003 | <0.0001 | <0.0001 |
| Women | 20.2 (0.8) | 21.0 (0.8) | 25.4 (0.8) | 26.9 (0.9) | 28.9 (0.9) | 32.7 (1.0) | 34.2 (0.9) | 35.6 (1.0) | <0.0001 | <0.0001 |  | <0.0001 | <0.0001 |
| Age (years) |  |  |  |  |  |  |  |  |  |  |  |  |  |
| 18-44 | 7.3 (0.4) | 8.9 (0.5) | 11.2 (0.5) | 13.5 (0.7) | 14.8 (0.7) | 16.4 (0.8) | 20.3 (0.8) | 21.2 (1.0) | <0.0001 | <0.0001 | <0.0001 | <0.0001 | <0.0001 |
| 45-64 | 17.1 (1.0) | 16.9 (0.9) | 22.7 (1.0) | 24.9 (1.0) | 26.1 (1.0) | 31.2 (1.1) | 31.6 (0.9) | 34.1 (1.1) | <0.0001 | <0.0001 |  | <0.0001 | <0.0001 |
| 65-118 | 22.3 (1.9) | 23.4 (1.7) | 30.0 (1.8) | 29.2 (1.7) | 32.3 (1.7) | 35.2 (1.7) | 36.2 (1.5) | 39.3 (1.5) | <0.0001 | <0.0001 |  | <0.0001 | <0.0001 |
| Region |  |  |  |  |  |  |  |  |  |  |  |  |  |
| Urban | 15.6 (0.9) | 13.9 (0.8) | 17.1 (0.8) | 18.1 (0.9) | 19.9 (1.0) | 21.4 (1.0) | 24.1 (0.9) | 28.2 (1.3) | <0.0001 | <0.0001 | 0.0001 | <0.0001 | <0.0001 |
| Rural | 10.4 (0.5) | 12.4 (0.5) | 16.4 (0.6) | 18.1 (0.6) | 18.8 (0.7) | 22.6 (0.7) | 24.5 (0.7) | 24.4 (0.8) | <0.0001 | <0.0001 |  | <0.0001 | <0.0001 |
| Education |  |  |  |  |  |  |  |  |  |  |  |  |  |
| Less than high school | 17.0 (0.8) | 17.7 (0.8) | 22.8 (0.8) | 24.5 (1.0) | 25.3 (1.0) | 30.7 (1.1) | 31.3 (1.1) | 32.9 (1.4) | <0.0001 | <0.0001 | 0.0015 | <0.0001 | <0.0001 |
| Highschool | 8.4 (0.8) | 8.8 (0.8) | 12.2 (0.8) | 15.2 (0.8) | 16.0 (0.9) | 18.8 (0.9) | 21.4 (1.0) | 24.6 (1.3) | <0.0001 | <0.0001 |  | <0.0001 | <0.0001 |
| University | 10.1 (1.1) | 9.8 (1.0) | 13.7 (1.0) | 15.3 (1.0) | 16.2 (1.0) | 17.7 (1.1) | 20.9 (0.9) | 20.8 (1.1) | <0.0001 | <0.0001 |  | <0.0001 | <0.0001 |

SE, standard error

^*^ P value for trend analysis. Logistic regression model was used without any adjustment in the model.

^⁑^ P value for trend analysis in which age, sex, region, and educational attainment were adjusted in the linear models except when used as a stratified variable.

^†^P value for comparison of 1993 and 2015. Chi-square test was used to compare differences. Bonferroni method was used for adjusting for multiple comparisons.

^‡^P value for comparison of 1993 and 2015 after adjusting for age, sex, region, and educational attainment except when used as a stratified variable.

**Supplementary Table 4 Age-standardized prevalence of central obesity among Chinese adults with BMI < 23 kg/m^2^**

|  | 1993 | 1997 | 2000 | 2004 | 2006 | 2009 | 2011 | 2015 | *P for trend | ^⁑^P for trend | P for interaction | ^†^P (1993 vs 2015) | ^‡^P (1993 vs 2015) |
| --- | --- | --- | --- | --- | --- | --- | --- | --- | --- | --- | --- | --- | --- |
|  | % (SE) | % (SE) | % (SE) | % (SE) | % (SE) | % (SE) | % (SE) | % (SE) |  |  |  |  |  |
| Total | 7.5 (0.4) | 8.1 (0.4) | 10.3 (0.4) | 11.8 (0.5) | 11.6 (0.5) | 14.3 (0.6) | 15.1 (0.5) | 17.4 (0.7) | <0.0001 | <0.0001 |  | <0.0001 | <0.0001 |
| Men | 2.0 (0.3) | 2.6 (0.3) | 4.2 (0.4) | 5.0 (0.5) | 3.9 (0.4) | 5.2 (0.5) | 6.3 (0.5) | 8.5 (0.9) | <0.0001 | <0.0001 | 0.0151 | <0.0001 | <0.0001 |
| Women | 13.1 (0.7) | 13.7 (0.7) | 16.6 (0.8) | 18.5 (0.9) | 18.8 (0.9) | 22.6 (0.9) | 22.7 (0.9) | 24.7 (1.0) | <0.0001 | <0.0001 |  | <0.0001 | <0.0001 |
| Age (years) |  |  |  |  |  |  |  |  |  |  |  |  |  |
| 18-44 | 4.0 (0.3) | 5.6 (0.4) | 7.0 (0.5) | 8.5 (0.6) | 8.9 (0.7) | 10.1 (0.7) | 12.2 (1.6) | 13.5 (1.0) | <0.0001 | <0.0001 | <0.0001 | <0.0001 | <0.0001 |
| 45-64 | 10.8 (0.9) | 9.7 (0.8) | 12.6 (0.9) | 15.0 (0.9) | 14.6 (0.9) | 19.0 (1.0) | 18.7 (0.9) | 22.1 (1.1) | <0.0001 | <0.0001 |  | <0.0001 | <0.0001 |
| 65-118 | 15.2 (1.7) | 15.6 (1.6) | 20.6 (1.7) | 20.7 (1.6) | 21.2 (1.6) | 26.1 (1.7) | 24.9 (1.5) | 28.2 (1.6) | <0.0001 | <0.0001 |  | 0.0002 | 0.0003 |
| Region |  |  |  |  |  |  |  |  |  |  |  |  |  |
| Urban | 10.0 (0.8) | 8.0 (0.7) | 11.3 (0.8) | 11.4 (0.8) | 12.1 (0.9) | 13.3 (1.0) | 14.6 (0.9) | 20.5 (1.3) | <0.0001 | <0.0001 | 0.0196 | <0.0001 | 0.0182 |
| Rural | 6.5 (0.5) | 8.1 (0.5) | 9.7 (0.5) | 11.9 (0.6) | 11.3 (0.6) | 14.7 (0.7) | 15.4 (0.7) | 15.5 (0.8) | <0.0001 | <0.0001 |  | <0.0001 | <0.0001 |
| Education |  |  |  |  |  |  |  |  |  |  |  |  |  |
| Less than high school | 10.7 (0.7) | 11.3 (0.7) | 13.9 (0.8) | 15.7 (0.9) | 15.5 (0.9) | 20.4 (1.0) | 19.8 (1.0) | 22.3 (1.4) | <0.0001 | <0.0001 | 0.0068 | <0.0001 | <0.0001 |
| High school | 5.5 (0.8) | 4.9 (0.6) | 6.9 (0.8) | 9.7 (0.8) | 8.7 (0.8) | 11.6 (0.9) | 12.8 (0.9) | 15.0 (1.2) | <0.0001 | <0.0001 |  | <0.0001 | <0.0001 |
| University | 6.6 (1.1) | 4.6 (0.8) | 9.0 (1.0) | 10.2 (1.0) | 10.2 (0.9) | 11.2 (1.0) | 13.1 (0.9) | 15.0 (1.1) | <0.0001 | <0.0001 |  | <0.0001 | 0.0009 |

SE, standard error

^*^ P value for trend analysis. Logistic regression model was used without any adjustment in the model.

^⁑^ P value for trend analysis in which age, sex, region, and educational attainment were adjusted in the linear models except when used as a stratified variable.

^†^P value for comparison of 1993 and 2015. Chi-square test was used to compare differences. Bonferroni method was used for adjusting for multiple comparisons.

^‡^P value for comparison of 1993 and 2015 after adjusting for age, sex, region, and educational attainment except when used as a stratified variable.
